# The involvement of aquaporin 5 in the inflammatory response of primary Sjogren’s syndrome dry eye: potential therapeutic targets exploration

**DOI:** 10.3389/fmed.2024.1439888

**Published:** 2024-09-23

**Authors:** Lijuan Fu, Zihang Zhao, Shuang Zhao, Meiying Zhang, Xiaoming Teng, Liyuan Wang, Tiansong Yang

**Affiliations:** ^1^School of Clinical Medicine, Heilongjiang University of Chinese Medicine, Harbin, China; ^2^Ophthalmology Department, First Affiliated Hospital of Heilongjiang University of Chinese Medicine, Harbin, China; ^3^Graduate School, China Academy of Chinese Medical Sciences, Beijing, China

**Keywords:** aquaporin-5, Sjogren’s syndrome dry eye, inflammatory response, therapeutic target, lacrimal gland

## Abstract

Sjogren’s syndrome (SS) is a chronic autoimmune disease. Mainly due to the infiltration of lymphoplasmic cells into the exocrine glands, especially the salivary glands and lacrimal glands, resulting in reduced tear and saliva secretion. Reduced tear flow can trigger Sjogren’s syndrome dry eye (SSDE). Although the pathophysiology of SSDE xerosis remains incompletely understood, recent advances have identified aquaporin-5 (AQP5) as a critical factor in dysregulation of the exocrine gland and epithelium, influencing the clinical presentation of SSDE through modulation of inflammatory microenvironment and tear secretion processes. This review aims to explore AQP5 regulatory mechanisms in SSDE and analyze its potential as a therapeutic target, providing new directions for SSDE treatment.

## 1 Introduction

Sjogren’s syndrome (SS) is a systemic autoimmune disease that primarily targers exocrine glands, particularly the lacrimal and salivary glands, leading to symptoms such as dry eyes (xerophthalmia) and dry mouth (xerostomia) due to chronic lymphoplasmacytic infiltration (1, 2). SS is classified into primary SS (pSS) and secondary SS (sSS), with pSS occurring independently and sSS often associated with other autoimmune connective tissue diseases (3, 4). As a systemic condition, SS can also impact various organs, potentially causing severe complications such as keratitis, corneal dystrophy, and scleritis, which may result in significant visual impairment (5–9). Despite advancements in understanding the SS’s pathobiology, many cases of SS-related dry eye disease (SSDE) remain undiagnosed, posing challenges for effective diagnosis and treatment (10).

Recent research highlights immune system dysregulation, particularly the interferon (IFN) pathway and persistent B-cell activation, plays a central role in SS pathogenesis (11–13). Additionally, epithelial cell dysfunction has garnered attention, with inflammation in these cells being critical in reducing tear production in SSDE (14). Aquaporin 5 (AQP5), a key protein in exocrine glands, has emerged as an important factor in this process. Alterations in AQP5 distribution and expression may worsen SSDE by impairing the function of affected glands (15). This review explores the role of AQP5 in epithelial cell inflammation in SSDE and its potential as a therapeutic target.

## 2 Overview of SSDE

SSDE is mainly marked by dry eye or keratoconjunctivitis sicca (KCS), resulting from lacrimal gland dysfunction that disrupts the tear film and exacerbates chronic ocular surface inflammation (8). The symptoms of SSDE are diverse, including photosensitivity, erythema, itching, or foreign body sensation, are often worsen with prolonged activities that reduce blinking, such as reading, driving, and watching TV (16).

While the clinical manifestations of SSDE, such as blurred vision, are more severe than those of non-Sjögren’s syndrome dry eye (NSSDE), no clear clinical features currently differentiate SSDE from NSSDE (17). SSDE is marked by significant lymphocytic infiltration in the lacrimal and salivary glands, initiating an autoimmune response that results in the gradual destruction of these glands (18). This infiltration frequently results in the formation of ectopic germinal centers (GC) -like structures in the affected tissues, found in 18–59% of SS patients, which disrupts glandular structure, intensify local inflammation, and promote autoantibody formation, culminating in reduced tear secretion and severe ocular surface damage (19–23). Corsiero confirmed that these GC-like structures mainly drive the risk of lymphoproliferative disease by promoting prolonged B-cell activation and clonal expansion (24).

The destruction of glandular tissue leads to tear film instability and triggers an inflammatory cascade within epithelial cells through pathways such as MAPK and NFκB. This cascade leads to the release of pro-inflammatory cytokines, which sustain not only the inflammatory state but also induce apoptosis of secretory acinar cells, leading to reduced production of the aqueous component of the tear film (25–27). Additionally, meibomian gland dysfunction and the apoptosis of goblet cells and epithelial cells compromise the lipid and mucin layers of the tear film, leading to increased tear evaporation and decreased ocular surface wettability (9, 28). Neurogenic inflammation and corneal nerve disorders further contribute to the vicious cycle of tear deficiency and chronic ocular surface inflammation by diminishing corneal sensation and impairing the blinking reflex (25).

**Figure 1:**
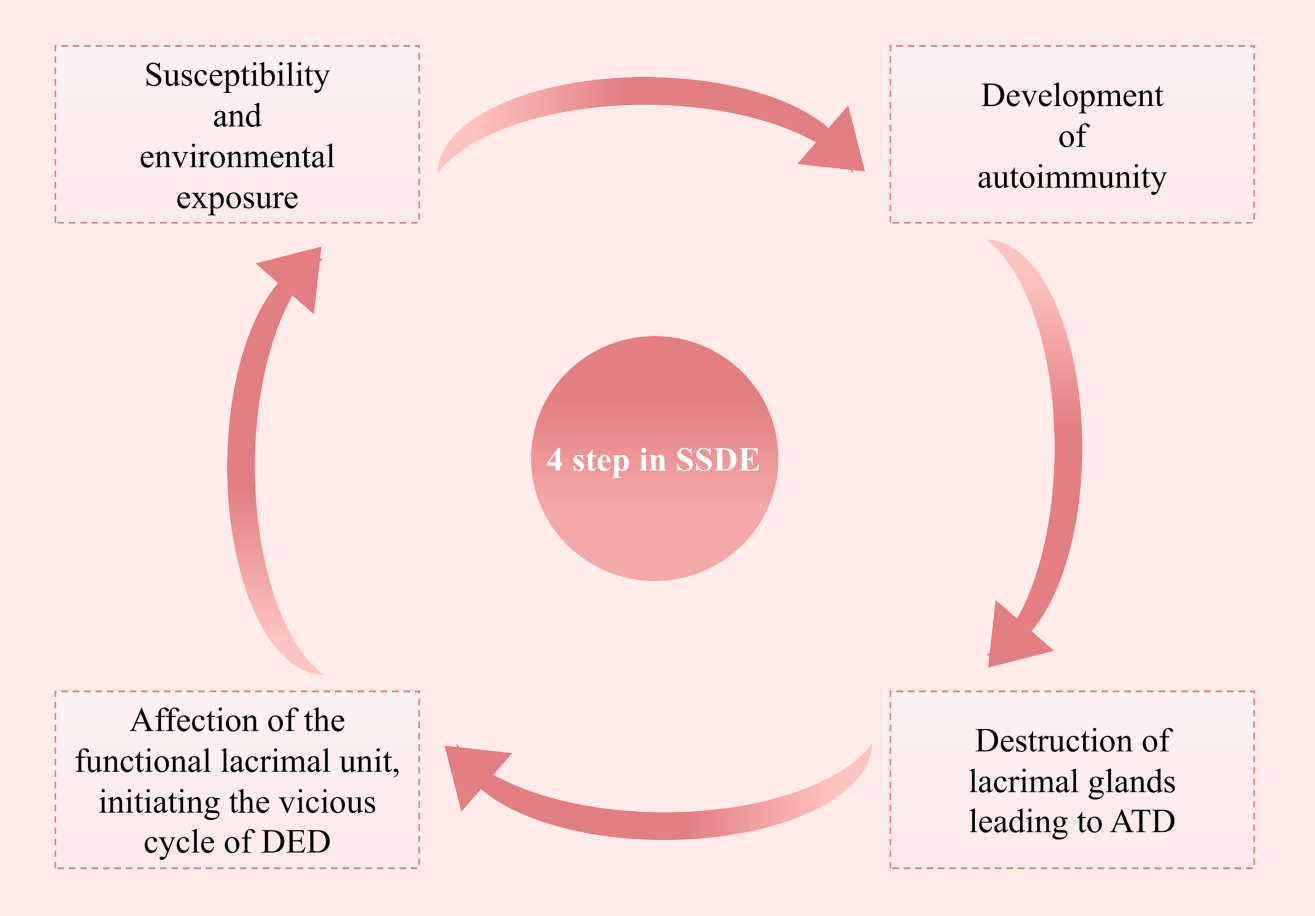
Four steps in the pathological process of SSDE. This figure outlines the four key steps involved in the pathological process of primary Sjögren’s Syndrome Dry Eye, highlighting the progression from initial susceptibility to the establishment of a chronic dry eye state (SSDE, Sjögren’s Syndrome Dry Eye; ATD, Aqueous Tear Deficiency; DED, Dry Eye Disease).

The pathogenesis of SSDE is intricate, and some researchers have summarized it into four stages (Figure 1) (29):

1. Interaction between genetic susceptibility and environmental exposure.
2. Development of autoimmunity.
3. Destruction of lacrimal glands leading to aqueous tear deficiency.
4. Affection of the functional lacrimal unit, initiating the vicious cycle of dry eye disease (DED).

## 3 Aquaporin 5

Aquaporins (AQPs) are membrane proteins that efficiently facilitate the rapid transport of water and small molecules across cellular membranes (30). To date, 13 AQP subtypes have been identified in humans and categorized into three subfamilies based on functional and structural similarities. The first group consists of classical water-selective AQPs (AQP0, AQP1, AQP2, AQP4, AQP5, AQP6, and AQP8), which primarily function as water transport channels. The second group includes glycerol channel proteins (AQP3, AQP7, AQP9, and AQP10), which transport not only water but also other small uncharged molecules such as ammonia, urea, and particularly glycerol. In some literature, these glycerol channels are also referred to as the “heretical” AQPs (31). The final subfamily, known as superaquaporins (AQP11 and AQP12), facilitates the transport of glycerol, water, and hydrogen peroxide (H_2_O_2_) (32). These channels exhibit minimal homology with the other subfamilies and are found exclusively in multicellular organisms, excluding fungi and plants (33).

Aquaporin-5 (AQP5) is a crucial member of the aquaporin family, playing a pivotal role in facilitating cellular water transport and participating in various cellular processes (34). AQP5 is widely expressed in diverse human tissues, particularly in the salivary and lacrimal glands, making it highly significant in the context of SSDE (35–39). Composed of 256 amino acids, AQP5 functions as a tetramer consisting of four identical monomers. Each monomer forms a water pore with six transmembrane alpha helices connected by five loops, with both the amino and carboxyl ends located in the cytoplasm. The folding of the B and E loops creates a half-helix that surrounds two highly conserved NPA motifs (asparagine-proline-alanine) (40, 41), which are situated in the narrow central constriction region of the channel and are crucial for ensuring the high selectivity of water and solutes through the pores (41). The transport function of AQP5 is regulated by various independent mechanisms, including phosphorylation at the Ser156 site, protein kinase A activity, and extracellular osmotic pressure (42).

The principal role of AQPs is closely linked to their molecular configuration. These monomers assemble into functional units, each with an independent water transport pathway, functioning autonomously within the AQP tetramer (43). By facilitating water permeability across the plasma membrane, AQPs enable the flow of intracellular fluid (44). Furthermore, post-translational modifications such as phosphorylation, ubiquitination, glycosylation, subcellular localization, degradation, and protein interactions play a critical role in regulating the physiological functions of AQPs.

The translocation of AQP from intracellular vesicles to the apical membrane of the cell is a complex process triggered by a combination of various stimuli, including post-translational modifications, neurotransmitter binding to G protein-coupled receptors (GPCRs), and interactions with multiple partner proteins along microtubules or within the membrane scaffold (45–47). This process regulates the transmembrane transport of water and small molecules. AQP2, a renal aquaporin, has been identified as a key player in renal water reabsorption following vasopressin stimulation. Due to the significant sequence similarity between AQP5 and AQP2, numerous studies have focused on elucidating the transport mechanism of AQP2 to lay the groundwork for understanding AQP5 translocation (48–50). Current knowledge of AQP5 transport in the salivary glands suggests that para-acetylcholine and norepinephrine must bind to M3 muscarinic and β-adrenergic receptors, respectively, to trigger an AQP5 response and facilitate its transfer to the apical membrane (51, 52).

Similarly, AQP5 exhibits a regulatory mechanism in the transport processes within the salivary and lacrimal glands. Upon stimulation by neuropeptides, AQP5 translocates from intracellular vesicles to the apical membrane, thereby enhancing water transport for secretions and facilitating saliva or tear secretion (52). Although two consensus PKA sites are present in loop D (Ser156) and the carboxyl-terminal (Thr259) of AQP5’s cytoplasmic structure, their phosphorylation does not directly influence protein transport (53). Instead, it has been suggested that regulation involves three independent mechanisms: phosphorylation at Ser156, protein kinase activity, and extracellular tonicity (42). However, further research is necessary to fully elucidate the molecular pathways governing AQP5’s translocation and function in tear secretion, especially in the context of SSDE. A deeper understanding of these mechanisms could provide valuable insights into therapeutic strategies aimed at restoring proper AQP5 function and alleviating symptoms in SSDE patients.

## 4 The expression of aquaporin 5 in the lacrimal glands

The lacrimal gland (LG) is a crucial component of the lacrimal duct system, primarily responsible for tear production and secretion, maintenance of ocular moisture, provision of essential nutrients, and cleansing and protection of the eye. The human LG consists of two distinct parts: the principal LG, located in the anterolateral lacrimal fossa at the orbit apex, and the accessory LG, found in the superior fornix of the eyelid. The primary LG is composed of three key elements: acinar cells, ductal units, and myoepithelial cells. The Lacrimal Gland Functional Unit (LFU) predominantly regulates tear generation, transportation, and elimination to maintain ocular homeostasis (54–56). The LFU includes primary and accessory lacrimal glands, meibomian glands, conjunctival goblet cells, surface epithelium, eyelids, the lacrimal drainage system, the glandular and mucosal immune systems, and interconnected innervation (57). The tear film consists of an aqueous mucin layer, overlaid by a lipid layer containing liquid and soluble components produced by the lacrimal gland and mucins secreted by goblet cells. The combination of water and mucin forms a single layer known as the mucinous hydrogel or mucinous water layer (58). Tears contain a diverse array of lipids, proteins, and glycoproteins that synergistically maintain ocular surface cleanliness, lubrication, and stability and are produced at an average daily rate of approximately 5 mL (58).

AQPs are widely distributed across the plasma membranes of various cell types, including the lacrimal glands, and play a pivotal role in water and tear secretion. AQP5, in particular, is predominantly found in the ductal epithelial cells and apical membranes of acinar cells within the human lacrimal gland, where it regulates primary saliva and tear production (59–61). The proper localization and function of AQP5 are crucial for the normal physiological activity of these glands. Studies have demonstrated that AQP5’s distribution varies depending on individual’s health. In healthy individuals or those with NSSDE, AQP5 is primarily localized to the apical surface of acinar cells. However, in patients with SSDE, AQP5’s distribution is more diffuse, suggesting that abnormal localization may contribute to reduced tear secretion (62, 63). This altered distribution of AQP5 has been further supported by knockout mouse models, where the absence of AQP5 leads to impaired salivary gland function, underscoring its critical role in exocrine gland physiology (62).

In the human lacrimal gland, AQP5 is predominantly expressed in ductal epithelial cells and is primarily localized to the apical membrane of acinar cells. AQP5 exhibits similar apical membrane localization in the lacrimal glands of rats (64, 65), mice, humans (66), and rabbits (67). However, there are variations in its expression levels across different species. For instance, in rat lacrimal glands, AQP5 proteins are mainly localized to the ducts and endothelial cells of acini, where they are more frequently expressed than other AQPs (68). Immunolabeling studies have revealed the presence of AQP5 on the basolateral membranes of both ductal and acinar cells in mouse lacrimal glands (69). Additionally, in rabbit lacrimal glands, AQP5 levels are significantly higher than AQP4 levels by orders of magnitude in acinar cells, as well as in interlobular and intralobular ducts (70). Another study found that AQP5 is present in both acinar and ductal epithelial cells at the tip of the lacrimal gland in healthy dogs (71).

LG functions similarly to other exocrine glands. Active ion pumping into the acinar cells initiates passive osmosis, which drives water flow to generate tight isotonic ultrafiltration (72). The epithelial cells further stimulate the secretion of fluid within the acinar cavity, facilitating the release of electrolytes and other substances, resulting in the production of an isotonic fluid rich in potassium and chloride (73). Tear secretion begins with the production of primary secretion at the secretory tip or acinus, which is then modified into the final secretion as it passes through the ductal system (73).

Tear production is tightly regulated by the nervous system and both basolateral and apical aquaporins may play a role in s water movement and tear formation within the LG. In rat salivary glands, AQP8 is present in basolateral acinar cells, while AQP5 is found in apical acinar cells both are involved in the passage of water during primary saliva production (74). Reduced expression of AQP5 has been correlated with impaired salivary gland function in samples from patients with Sjögren’s syndrome (75). The neural response package stimulates the efferent sympathetic and parasympathetic neural networks innervating the LG, as well as the sensory nerves of the cornea and conjunctiva. Activation of the parasympathetic and sympathetic nerves leads to the release of neurotransmitters, which in turn triggers the secretion of water, electrolytes, and proteins from the surface of the LG and the eye (76). In BALB/c mice, unilateral sympathetic nerve transection resulted in bilateral LG iron death, down-regulation of AQP5, and decreased tear secretion through the VIP/Hif1a/TfR1 pathway (77).

## 5 Inflammation plays a crucial role in the involvement of AQP5 in SSDE

**Figure 2:**
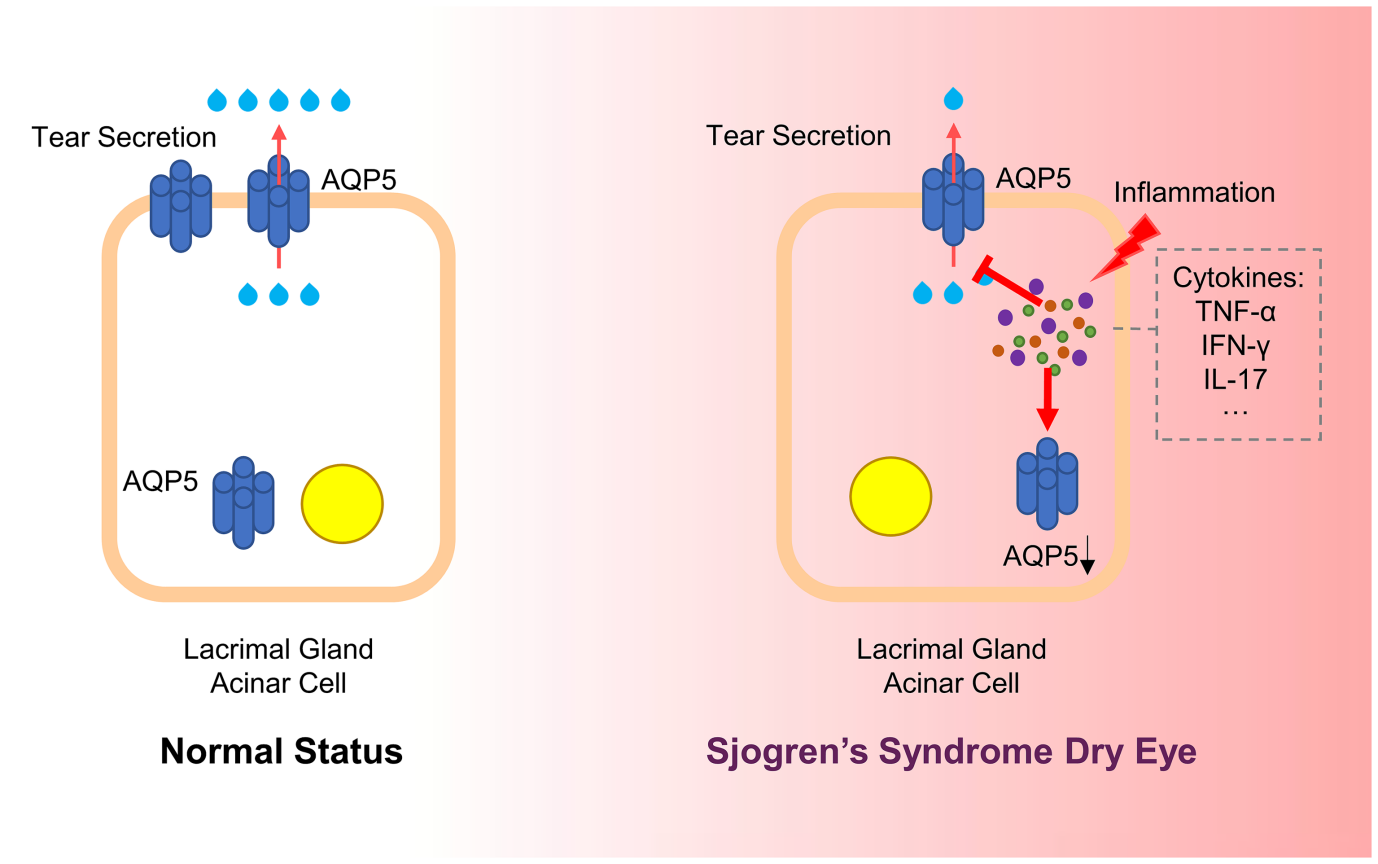
Inflammation affects AQP5 in SSDE. In Sjögren’s Syndrome Dry Eye, chronic inflammation disrupts AQP5 expression and localization. Inflammatory cytokines, such as TNF-α, IFN-γ, and IL-17, are released in response to an autoimmune attack on the lacrimal glands. These cytokines interfere with AQP5 function by down-regulating its expression and causing its mislocalization from the apical to the basolateral or cytoplasmic regions of the cell. This disruption impairs water transport across the acinar cells, reducing tear secretion and contributing to ocular dryness (AQP5, Aquaporin 5; SSDE, Sjögren’s Syndrome Dry Eye; TNF-α, Tumor Necrosis Factor-alpha; IFN-γ, Interferon-gamma; IL-17, Interleukin-17).

AQP5 plays a crucial role in fluid secretion within exocrine glands, and its dysregulation is closely associated with the pathogenesis of SSDE. Numerous studies have emphasized the impact of inflammation on AQP5 expression and localization, which significantly contributes to the hallmark symptoms of dry eye observed in SSDE (78, 79). Inflammation has been shown to alter AQP5 expression in the lacrimal glands, leading to impaired secretion. This abnormal expression of AQP5 in SSDE is believed to be a direct result of inflammation (Figure 2). For instance, studies have demonstrated that blocking pro-inflammatory cytokines like IL-7 or administering anti-TNF antibodies can restore AQP5 levels and improve symptoms in SS models, suggesting a strong connection between inflammation and AQP5 dysregulation (80, 81).

The nuclear factor kappa-light-chain-enhancer of activated B cells (NFkB) plays a dual role in regulating AQP5 during inflammation (82). NFkB can either enhance AQP5 mRNA levels in response to hyperosmolar stress or inhibit AQP5 transcription through interactions with other inflammatory mediators (83). Additionally, the proper trafficking and localization of AQP5 are dependent on interactions between prolactin-induced protein (PIP) and the ezrin domain with AQP5’s C-terminus (84). Disruption of these interactions due to inflammation leads to abnormal AQP5 distribution, which is associated with glandular dysfunction and reduced tear secretion.

In patients with pSS, autoantibodies targeting AQP5 have been detected, which are closely linked to decreased salivary secretion, suggesting a direct impact on glandular function (85, 86). Malfunction in AQP5 transport is hypothesized to contribute to SSDE, where the binding of anti-M3R autoantibodies hinders the transport of AQP5 to membrane units, thereby disrupting normal secretory processes (62). In SS mouse models, AQP5 shows an affinity for a 21 kDa protein absent in normal controls, whereas normal mice express a 17 kDa AQP5-binding protein not present in SS models, highlighting differences in AQP5 interaction and localization under pathological conditions (87).

Furthermore, AQP5 leakage into tears is observed in SS patients due to the destruction of lacrimal acinar cells by lymphocyte infiltration. This leakage also noted in animal models with dacryoadenitis but not in normal mice, suggests a strong correlation between the presence of AQP5 in tears and lacrimal gland injury (87, 88). Additionally, studies in rabbits with autoimmune dacryoadenitis have shown a decrease in AQP5 expression in acinar cells and an increase in ductal cells, indicating that specific duct segments may play a significant role in tear secretion (70).

Inflammation has been shown to induce significant changes in AQP5 localization. In NOD mice, for example, AQP5 is predominantly expressed on the apical membrane of acinar cells in younger, inflammation-free animals. However, in older mice with pronounced inflammation, there is a marked reduction in apical AQP5 expression and a corresponding increase in basolateral expression, suggesting that inflammation directly alters AQP5 distribution (89, 90). Similar findings have been corroborated in other mouse models, including E2f1−/− mice and specific IA phosphoinositol three kinase knockout mice, further supporting the role of inflammation in disrupting AQP5 localization (90–92).

The relationship between inflammation and aberrant AQP5 localization is further supported by studies showing a decrease in apical AQP5 and an increase in basolateral and cytoplasmic localization in various mouse models. These changes are believed to result from inflammatory infiltration and damage to glandular epithelium, underscoring the role of the inflammatory environment in disrupting AQP5 transport (90). Treatment with TNF-α has been observed to downregulate AQP5 in human salivary gland acinar cells, while the injection of TNF-α antibodies in NOD mice reduced glandular lesions and increased the expression of tight junction proteins and AQP5 (93). Additionally, the neutralization of IFN-γ in NOD mice treated with anti-PDL1 improved AQP5 expression and salivary secretion, supporting the hypothesis that inflammation plays a significant role in AQP5 dysregulation (94).

Therapeutic interventions targeting inflammation have shown promise in restoring AQP5 function. In SS mouse models, the delivery of an adeno-associated virus (AAV2)-AQP1 vector or the correction factor C18 for the cystic fibrosis transmembrane conductance regulator was found to resolve inflammation and restore saliva flow (95, 96). Similarly, blocking IL-17 attenuated inflammation and enhanced salivary secretion, while the administration of vasoactive intestinal peptide reduced IL-17 expression, increased AQP5 expression, and restored salivary secretion (97, 98).

**Figure 3:**
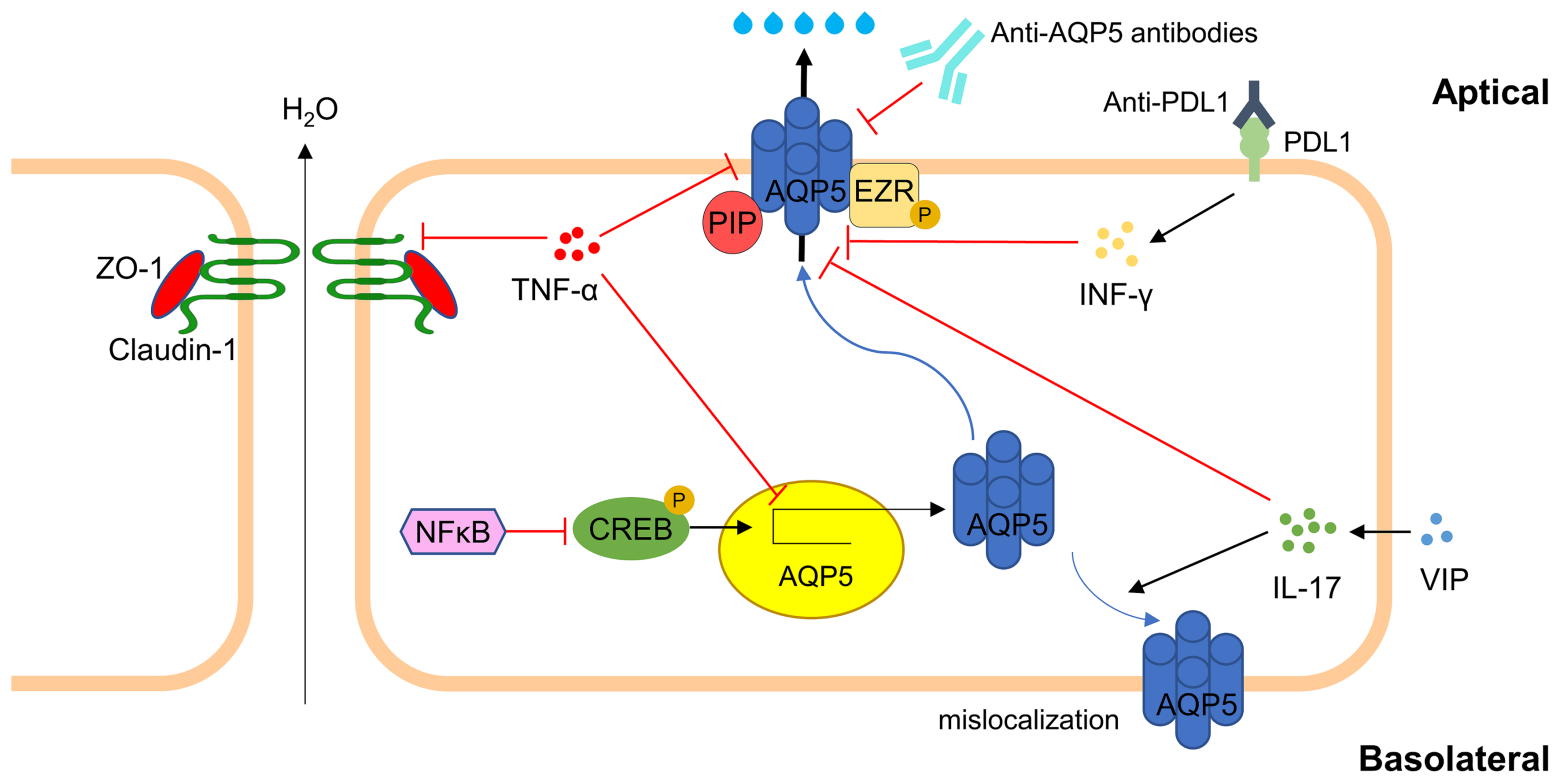
Major inflammatory molecular pathways involving AQP5 in SSDE. In Sjögren’s Syndrome Dry Eye, inflammation leads to the dysregulation of AQP5. TNF-α interfere with tight junction integrity (e.g., ZO-1, Claudin-1) and down-regulate AQP5 translation via NFkB/CREB pathway. Autoantibodies against AQP5 and PDL1 further exacerbate AQP5 dysfunction. Regulatory molecules like VIP can partially affect AQP5 expression and localization. The disruption of AQP5 affects tear production, aggravating dry eye symptoms in SSDE (AQP5, Aquaporin 5; TNF-α, Tumor Necrosis Factor-alpha; IFN-γ, Interferon-gamma; IL-17, Interleukin-17; NFkB, Nuclear Factor kappa-light-chain-enhancer of activated B cells; CREB, cAMP Response Element-Binding Protein; VIP, Vasoactive Intestinal Peptide; ZO-1, Zonula Occludens-1; PDL1, Programmed Death-Ligand 1; PIP, prolactin-induced protein; EZR, ezrin).

The integrity of acini and the proper expression of tight junctions between epithelial cells are essential for establishing apicobasal polarity and regulating the paracellular flow of ions and water (99). In SS, pro-inflammatory cytokines have been demonstrated to disrupt the polarity of acinar cells in exocrine glands by inducing the apical reorientation of proteins responsible for maintaining cell polarity (100–102). Consequently, the aberrant localization of AQP5 in acinar cells of the salivary or lacrimal glands may result from decreased or absent expression of cell polarity proteins linked to local pro-inflammatory cytokine production (63, 103). Other mechanisms, such as abnormal B lymphocyte hyperreactivity and the production of autoantibodies against M3 muscarinic receptors and anti-AQP5 antibodies, may also contribute to the altered distribution of AQP5 and the hypofunction of the salivary or lacrimal glands. The presence of anti-AQP5 antibodies in patients with pSS is correlated with reduced AQP5 function, decreased secretion flow, and histopathological changes in the secretory glands (104–106). Mice immunized with peptides derived from melanin Prevotella AQP5 homologous AQP, produce anti-AQP5 antibodies, which subsequently lead to reduced salivary flow (107). Overall, inflammation and autoimmunity play critical roles in the dysregulation of AQP5 in secretory glands, contributing to the diminished secretory efficiency observed in SSDE. AQP5’s involvement in these processes, influenced by various inflammatory molecular pathways is illustrated in Figure 3.

## 6 Potential therapeutic value of AQP5

The current approach to treating SSDE primarily focuses on alleviating the “fatigue-pain-dryness” triad of symptoms, however, the outcomes remain unsatisfactory. Initial pharmacological treatments for SSDE is centered around artificial tears and lubricating ointments, which mainly address symptoms rather than the underlying causes (108). Due to potential complications, such as cataracts and glaucoma, the use of steroids should be limited to short-term treatment in patients who have not responded adequately to other therapies (8). Pilocarpine, an oral medication that stimulates lacrimal and salivary gland secretion, has shown efficacy in alleviating dry eye symptoms and may also improve objective measures of dry eye (109). For patients who are resistant to medication and maximal lubrication may benefit from lacrimal occlusion. Local autologous serum can also be employed to treat ocular surface injuries, particularly in case of severe dry eye and corneal ulcers (8).

Recent studies have demonstrated the potential of targeting AQP5 as a therapeutic strategy for SSDE. For instance, ambroxol, a mucolytic agent, has been shown to upregulate AQP5 and MUC5AC mRNA and protein expression levels in goblet cells, suggesting its potential in enhancing tear secretion and treating SSDE by modulating AQP5 (110). Neuropathic ophthalmia is a prevalent symptom of moderate to severe dry eye, and gabapentin (GBT) has been particularly effective in managing ocular surface nerve pain in dry eye conditions due to its analgesic, anti-inflammatory, and secretory properties. It can enhance the expression of acetylcholine and norepinephrine and induce AQP5 expression in lacrimal glands, thereby alleviating tear irritation (111). Increased tear secretion effectively reduces friction on the ocular surface, making this a valuable mechanism for SSDE therapy. Additionally, PACAP eye drops have been shown to elevate AQP5 levels in the LG acinar membrane, while treatment with AQP5 siRNA significantly decreases tear production induced by PACAP.

Natural compounds targeting AQP5 have also been explored for their potential to modulate glandular secretion abnormalities in SS patients. For example, ginsenoside Rb1 activates AQP5 transcription by binding to estrogen receptor α, significantly enhancing saliva secretion, while Dendrobium extract improves glandular secretion and alleviates dryness symptoms by modulating AQP5 expression (112). The regulatory effects of these natural compounds on AQP5 suggest their potential utility in treating SSDE. Interestingly, certain non-pharmacological complementary and alternative therapies have also demonstrated a regulatory effect on AQP5 in SSDE patients. Some researchers have proposed the use of prebiotics, such as highly bioavailable polyphenols, to modulate the gut microbiota in SS patients as an adjunctive treatment for dry eyes and dry mouth (113). These findings suggest that natural compounds and alternative therapies may provide complementary strategies for managing SSDE by targeting AQP5.

Gene therapy presents a promising avenue to overcome the limitations of traditional therapies, particularly for SSDE. With advancements in gene transfection technology, gene therapy for lacrimal glands has become increasing feasible. For instance, delivering the AQP1 gene to the salivary glands of radiation-affected rats and miniature pigs has been demonstrated to enhance saliva flow (114, 115). Moreover, clinical trials have demonstrated that introducing the AQP1 gene into the submandibular glands of patients improves salivary gland secretion. Although no clinical trials have yet been conducted on AQP5 virus vector transfection for SSDE, this approach holds potential as a future treatment for enhancing tear secretion in SSDE (116).

## 7 Conclusion

AQP5 plays a pivotal role in regulating of lacrimal gland membrane permeability and is essential for tear production. The dysregulation of AQP5 in SSDE is strongly influenced by the inflammatory microenvironment, systemic exogenous factors, and interactions between lymphocytes and epithelial cells, as evidence in both patients and animal models. Furthermore, significant alterations in AQP5-related protein interactions may underlie some of the pathological features observed in SS patients. Despite the crucial role of AQP5 in SSDE, therapeutic strategies specifically targeting AQP5 are still limited. Although there have been efforts to restore salivary secretion by promoting AQP5 transport to the acinar apical membrane, achieving complete restoration of AQP5 localization and function remains a significant challenge.

Future research should focus on deepening our understanding of the mechanisms through which inflammatory cytokines and immune cells contribute to AQP5 dysregulation, as these pathways could serve as potential therapeutic targets. Nonetheless, it is essential to critically evaluate the existing literature to balance the strengths and limitations of current studies. While the therapeutic potential of targeting AQP5 in SSDE is substantial, there are inherent challenges in translating these findings into effective treatments. Continued research aimed at refining AQP5-targeted therapies, along with a personalized treatment approach, will be crucial in developing more effective options for patients with SSDE.
